# Sedentary lifestyle and Framingham risk scores: a population-based study in Riyadh city, Saudi Arabia

**DOI:** 10.1186/s12872-019-1048-9

**Published:** 2019-04-08

**Authors:** AlJohara M. AlQuaiz, Amna Rehana Siddiqui, Ambreen Kazi, Mohammad Ali Batais, Ali M. Al-Hazmi

**Affiliations:** 1 Princess Nora Bent Abdallah Research Chair for Women Health Research, Deanship of Research Chairs program, King Saud University, Riyadh, Kingdom of Saudi Arabia; 2 Department of Family & Community Medicine, College of Medicine, King Saud University, Riyadh, Kingdom of Saudi Arabia

**Keywords:** Framingham risk scores, cardiovascular disease risk, Physical inactivity, Sitting time, Waist circumference

## Abstract

**Background:**

Studies from Saudi Arabia have reported a continued increase in the prevalence of cardiovascular diseases and their associated risk factors. The objective of this study was to measure the gender differences in the cardiovascular disease (CVD) risk based on Framingham risk scores (FRS) and to explore the association of FRS with sedentary life style including physical inactivity, sitting time and central obesity among Saudi adults.

**Methods:**

A cross-sectional survey was conducted on 2997 Saudi adults (males = 968, females = 2029) selected from 18 primary health care centres in Riyadh city, from December 2014 to August 2015. A detailed interview that evaluated lifestyle and past medical history was conducted; furthermore, anthropometric measurements and blood samples were collected for lipid profiling. The FRS were calculated based on the age, gender, systolic blood pressure, treatment for hypertension, diabetes, smoking status, total blood cholesterol and high-density lipoprotein levels. These scores were categorized into low risk (FRS < 10) and high/intermediate risk (≥10). A multivariable logistic regression analysis was performed.

**Results:**

The mean (±SD) age of the males and females was 43.1(±11.7) vs 43.8(±10.9) years (*p* = 0.07), respectively. The number of Saudi male participants with intermediate-to-high FRS scores (≥10) was almost twice that of females (males 33% vs 17%). The multivariable logistic regression model after adjusting for education level and housing type, found that *low physical activity* (aOR & 95%CI for males 2.91 (1.45, 5.80); females 1.38 (1.06, 1.81); *prolonged sitting time* (aOR &95%CI for males 1.36 (0.98, 1.90) females 1.58 (1.20, 2.07), high *central obesity* (defined as waist circumference in males > 102 cms, and females > 88 cms) (aOR & 95%CI for males 2.38 (1.67, 3.41); females 3.35 (1.92, 5.87) were associated with high/ intermediate risk for CVD.

**Conclusions:**

A significant percentage of Saudi population revealed FRS ≥10. Females beyond the age of 50 were found to have a higher prevalence for CVD risk compared with males of the same age group. Modifiable risk factors like low physical activity, prolonged sitting time and central obesity have strong implications for primary prevention and management services that can change the risk profile of the Saudi population.

**Electronic supplementary material:**

The online version of this article (10.1186/s12872-019-1048-9) contains supplementary material, which is available to authorized users.

## Background

Cardiovascular disease (CVD) accounts for 31% of all global deaths, and more than 18 million people die from CVD-related causes annually [1]. CVD is an increasing public health concern in the Middle East and the Gulf Council Countries (GCC) [2–4]. It is estimated that the overall deaths from CVD in the GCC countries, including Saudi Arabia, represent over 45% of all deaths [1]. It is projected that the prevalence of CVD in the Kingdom of Saudi Arabia (KSA) will rapidly increase in the future [5].

Different International studies, such as The INTERSTROKE and INTERHEART studies [6, 7], the Gulf Registry of acute coronary events (Gulf RACE) [2] and the Africa Middle East Cardiovascular Epidemiological (ACE) study [8] have, all recognized a common set of risk factors that are associated with CVD. These factors include hypertension, diabetes mellitus, dyslipidaemia, obesity, smoking, physical inactivity, poor dietary habits, and alcohol consumption [2]. The modifiable risk factors, collectively accounted for 90 and 94% population attributable risk in males and females, respectively [7]. Studies from Saudi Arabia have also reported that the prevalence of these risk factors are on the rise during each consecutive year [9–11]. Varying results on gender differences have been reported by the previous research studies in context to CVD prevalence and the associated factors. [2–4]. The prevalence of CVD has been found to be higher in males as compared to the females [2–4]. Majority of the studies report that factors like diabetes mellitus, hypertension, physical inactivity and smoking were more prevalent among the males as compared to the males. Whereas, overweight and obesity was more commonly reported among the females [2–11].

Over the past few decades, many studies have shown that individuals leading sedentary lifestyles have higher levels of CV risk factors and an increased risk of incident CVD [12, 13]. A study by Chau et al. demonstrated that adults who sit for ≥10 hours/day (h/day) had a 65% and 115% greater risk of overall and cardio-metabolic-related mortality, respectively, compared to those with a sitting time of < 4 h/day after adjusting for potential confounders (age, smoking, physical activity, body mass index, education level, health status and cardio-metabolic disease status with age) [12]. Similarly, another study demonstrated that a longer duration of total daily sitting time (> 10 h compared to < 6 h/day) can increase the risk of myocardial infarction (MI) by 38% and the overall risk of mortality by 31% [14]. In Saudi Arabia, physical inactivity received attention during the last decade with studies showing an alarmingly high number of adults (ranging from 75% to 90%) reporting less than sufficient levels of physical activity [15–18].

Many risk-assessment tools are available [19–24], which can objectively measure the impact of various CV risk factors and provide an estimate of CV risk [2, 24–26]. Subsequently, these can be used to guide the development of disease prevention strategies and management interventions for patients at a high risk of CVD [25, 26]. Among these tools, the Framingham risk score (FRS) is the most frequently used tool [21, 23]. The clinical practice guidelines for the primary prevention of coronary heart disease (CHD) also recommend a risk management approach based on the FRS [27]. The FRS is available in various application formats, such as point scoring systems, risk charts, or web-based calculators [28].

The Framingham heart study, conducted by Al Humaidi, in Abha province, KSA, have reported a mean risk of 8.9% for developing coronary artery disease [29]. Another recent study that was conducted among the military population, which were expected to have higher fitness level, revealed that one-tenth of the participants had a 10-year CVD risk of ≥10%, with a mean risk of 4.5% [30]. However, the probability of 10 year-CVD risk based on the FRS and its association with sedentary life style in the general Saudi population remains largely unknown. Hence, the objective of this study was to measure the gender differences in the probability of 10-year risk for CVD based on the FRS and to examine the association of FRS with sedentary lifestyles among Saudi males and females aged 30 to 75 years.

## Methods

### Study design and setting

This study was part of a large cross-sectional survey (Women in Saudi Arabia Health Examination Survey; WISHES), which aimed to measure the prevalence, severity and factors correlated with various chronic diseases in males and females aged 30 to 75 years in Riyadh city, Saudi Arabia. Data were collected between December 2014 to August 2015. There are 105 primary health care centres (PHCCs) in Riyadh city, out of which 18 were randomly (https://www.random.org/) selected from the five administrative regions of Riyadh. In addition to PHCCs, we approached five government institutions (technical institutes, college/university and social organizations) to enrol eligible male participants (because males were not able to attend PHCCs as most of them were at work day time).

### Study participants

Saudi adults between 30 and 75 years of age who were permanent residents of Riyadh city were eligible to participate in the WISHES study. Non-Saudis, pregnant women and those with cognitive impairment were not included in the study.

Initially, 3100 adults (1000 males and 2100 females) were invited to participate in the study. Among these adults, 975 males and 2038 females fulfilled the eligibility criteria and gave informed and signed consent. However, surveys from 17 of these individuals were discarded due to incomplete blood report/interviews; therefore, 2997 participants (968 males and 2029 females) were finally included in the analysis. The study protocol was approved by the Institutional Review Board (IRB), King Saud University (E-12-658) and the Institutional Review Board of the Ministry of Health, Dammam (IRB ID MOH0151).

### Data collection tools

A questionnaire was developed that comprised a detailed socio-demographic profile, past and current medical history, family history, smoking history and reproductive history (for females only). A copy of the questionnaire specific to this manuscript is attached as “Additional file 1”. A team of five females and two male phlebotomists/data collectors who were well-versed in the Arabic and English languages were rigorously trained by the researchers to conduct the interviews. A pilot study was conducted on a sample of 50 participants to pre-test the questionnaire and assess the feasibility for conducting the study.

### Anthropometric and blood pressure measurements

The anthropometric indices included weight, which was measured with an electronic scale (Secca 220—Hamburg, Germany, 2009), and height, which was measured using the standard method with a stadiometer [31]. Both, weight and height were used to calculate body mass index (BMI) as weight in kg divided by height in meters squared. Waist circumference (WC) was measured using a measuring tape at the mid-point between the lowest rib and top of the hip bone (iliac crest) [32]. Central obesity for the males and the females was defined as waist circumference of > 90 and > 80 cms, and high central obesity as > 102 and > 88 cms, respectively (WHO, 2008). Two blood pressure readings were taken using the oscillometric method with the participant in an upright position, according to the instruction manual (Omron-5 Series_TM Blood Pressure Monitor Model BP742—China 2010). The average of both readings was computed.

### Physical activity

We used the validated International Physical Activity questionnaire (IPAQ, short form) [33]. The items in the short IPAQ form are structured to provide separate scores for walking, moderate-intensity and vigorous-intensity activity. MET minutes/week (multiples of the resting metabolic rate) were calculated by multiplying the duration of PA (in minutes) with the number of days (per week) and further multiplying with pre-assigned metabolic values of 2.2, 4.0 and 8.0 for walking, moderate-intensity and vigorous-intensity activities, respectively [34]. Continuous scores were converted into the low, moderate and high physical activity categories according to the scoring guidelines [34]. Sitting time, which was considered an indicator of time spent in sedentary activity, was calculated as a continuous variable based on the average time spent sitting on a particular week day (both at work or at home).

### Framingham risk scores for CVD

The FRS covers the full spectrum of CVD, including coronary heart disease, peripheral vascular disease, stroke and heart failure [35]. The online FRS calculator is user-friendly and free of cost [36]. The online calculation requires information on the age, gender, systolic blood pressure (at the time of the interview), treatment for hypertension (yes/no), diabetes (yes/no), smoking (yes/no), total blood cholesterol and high-density lipoprotein (HDL) levels for each participant. The summation of these variables resulted in a continuous score for each participant using the FRS online calculator. These scores were further categorized as follows: individuals with low scores (< 10) were considered to have < 10% risk; those with intermediate scores (between 10 and 20) were considered to have 11 to 20% risk; and those with high scores (> 20) were considered to have > 20% risk of developing CVD in the next ten years [35].

### Blood collection procedures

A random sample of non-fasting 10 mL of venous blood was collected using a needle of 22 or 23 gauge along with a sample adaptor to fill the test tube (5 mL in a yellow-capped test tube for basic biochemistry [cholesterol, high density lipo-protein (HDL)]. The test tubes were placed into a labelled plastic bag, which was placed in a cold box lined with ice packs. Then, the samples were transferred to the laboratory of King Khaled University Hospital (KKUH) at the end of the working day and stored at a temperature of 2 to 8 degrees Celsius until analysis.

### Measurement of lipids

Serum levels of total cholesterol and HDL-cholesterol were measured with a fully automated analyser (Siemens Dimension RxL, Germany) using enzymatic methods. The intra-assay and inter-assay coefficients of variation were 0.84 and 1.30, respectively, for total cholesterol, and 1.9 and 2.1 for HDL-cholesterol.

### Statistical analysis

The data were analysed using the Statistical Package for the Social Sciences computer software package (IBM SPSS statistics version 21.0). The mean values and standard deviations were computed for the continuous variables, and frequency and proportions were calculated for the categorical variables. The Student’s t-test for independent samples and the Chi-squared test were used to analyse the differences between FRS and the associated variables in relation to gender. The Pearson’s correlation coefficient (r) was calculated between continuous variables. The outcome variable (FRS) was evaluated as a dichotomous variable for which participants with an intermediate or high FRS (≥10) were considered “at risk” and coded as (1), vs. low risk (FRS < 10), which was coded as (0). The multivariable logistic regression analyses were used to estimate the association between FRS (outcome variable) and sedentary lifestyle (exposure variable), including physical activity, sitting time, and waist circumference. The model was adjusted for education level and type of housing (proxy indicator for socioeconomic status). Vigorous and moderate physical activity were summed to create one variable (coded as 1) vs. low physical activity (coded as 0). Continuous sitting time of the participants was converted to hours and categorized according to average sitting time into a dichotomous variable coded as 0 = ≤6 h and 1= > 6 h [37]. The level of statistical significance was set as *p* < 0.05. All plausible interactions were checked before the development of the model. The Hosmer Lemeshow goodness of fit test was used to assess the model fit.

## Results

The number of Saudi male participants with intermediate-to-high FRS scores (≥10) was almost twice that of females (males 33% vs 17%). Similarly, the mean (±SD) FRS for the males were higher as compared to females (9.5(±8.6) vs 5.5 (±6.5) *p* < 0.001). Comparisons between age and gender revealed that a significant proportion of males between ages 30–50 years had FRS ≥10. Whereas, for females, a significant proportion between 51 and 75 years had FRS ≥10 (Table 1).

**Table 1:** Framingham risk scores in Saudi males and females, by age category (*n* = 2997)

Framingham Risk scores	Age in years^b^				Total = 2997^a^ Males = 968 Females = 2029
	30–40	41–50	51–60	61–75 years	
Low risk (score < 10)^1^					
*Males*	450 (69.4)	164 (25.3)	31 (4.8)	3 (0.5)	648 (66.9)
*Females*	929 (55.2)	513 (30.5)	209 (12.4)	32 (1.9)	1683 (82.9)
*Total*	1379 (59.2)	677 (29.0)	240 (10.3)	35 (1.5)	2331 (77.8)
Intermediate Risk (score ≥ 10–20)^1^					
*Males*	19 (10.4)	82 (44.8)	62 (33.9)	20 (10.9)	183 (18.9)
*Females*	2 (0.8)	55 (23.2)	123 (51.9)	57 (24.1)	237 (11.7)
*Total*	21 (1.5)	137 (32.6)	185 (44.0)	77 (18.3)	420 (14.0)
High Risk (score > 20)^2^					
*Males*	4 (2.9)	26 (19.0)	45 (32.8)	62 (45.3)	137 (14.2)
*Females*	1 (0.9)	5 (4.6)	43 (39.4)	60 (55.0)	109 (5.4)
*Total*	5 (2.0)	31 (12.6)	88 (35.8)	122 (49.6)	246 (8.2)

^1^*p* value < 0.001; ^2^*p* value < 0.01

^a^Column percentage; ^b^ Row percentage

**Fig. 1:**
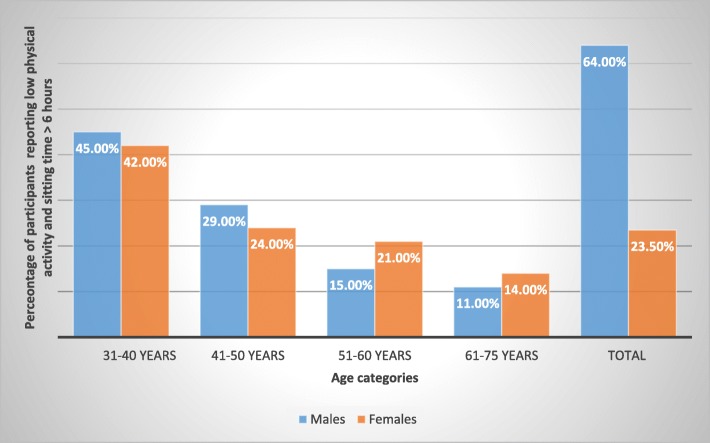
Percentage of males and females reporting low physical activity & sitting time of > 6 h by age categories in Riyadh, Saudi Arabia

The mean age between males and females was not significantly different [43.1 (± 11.7) vs 43.8(±10.9), *p* = 0.07)]. Males exhibited a higher mean waist circumference compared to females [96.2 ((±14.2) vs 92.7 (±14.0) (*p* < 0.001)], whereas the mean body mass index for females was higher than for males [29.6 (±6.4) vs 31.4 (±6.5) (p < 0.001)]. Significant correlation was observed between BMI and waist circumference (*r* = 0.4, *p* < 0.001) (results not shown). Males exhibited significantly lower mean HDL levels compared to females (Table 2). The age and gender comparison revealed that in the male participants, risk factors such as diabetes mellitus, hypertension, hypertriglyceridemia low HDL, smoking were more frequently reported/detected in the age groups of 30 to 50 years; whereas in females, a significant increase in these risk factors was observed in the age group of 51 to 60 years old (results not shown). Similar to above factors, physical activity and sitting time showed similar trend. The highest percentage of participants with low physical activity and prolonged sitting time belonged to 30–40 years age category, whereas after 50 years of age, more females reported sedentary life style compared to the males (Fig. 1).

**Table 2:** Descriptive characteristics under Framingham risk scores for Saudi males and females in Riyadh, Saudi Arabia, *N* = 2997

Variables	Males Mean (±SD)	Females Mean (±SD)	*P* value
Framingham Risk scores	9.4(±8.6)	5.4 (±6.5)	**< 0.001**
Age (in years)	43.1(±11.7)	43.8(±10.9)	0.07
Systolic Blood pressure (mmHg)	124(±15.2)	120(±18.0)	**< 0.001**
Mean Cholesterol levels (mg/dL)	194(±41.5)	195(±37.3)	0.2
Mean HDL levels (mg/dL)	43.1(±10.8)	54.1(±13.8)	**< 0.001**
Age (in years category) (n%)			
30–40	473 (48.9)	932 (45.9)	**0.02**
41–50	272 (28.1)	573 (28.2)	
51–60	138 (14.3)	375 (18.5)	
61–75	85 (8.8)	149 (7.3)	
Systolic Blood Pressure(mmHg)			
≤ 140 mmHg	824 (85.1)	1753 (86.4)	0.3
> 140 mmHg	144 (14.9)	276 (13.6)	
Treatment for Hypertension			
Yes	99 (10.2)	344 (17.0)	0.6
No	869 (89.8)	1685 (83.0)	
Diabetes Mellitus			
Yes	164 (16.9)	442 (21.8)	**0.02**
No	804 (83.1)	1587 (78.2)	
Smoking			
Yes	268 (27.7)	44 (2.2)	**< 0.001**
No	700 (72.3)	1985 (97.8)	
Blood Cholesterol level (mg/dL)^a^			
Normal	577 (59.6)	1180 (58.2)	0.4
Raised	391 (40.4)	849 (41.8)	
High Density Lipoprotein (mg/dL)^b^			
Normal	564 (58.3)	1744 (86.0)	**< 0.001**
Abnormal	404 (41.7)	285 (14.0)	

^a^Reference value for males and females was taken as normal ≤200 mg/dl and raised> 200 mg/dl

^b^Reference value for males and females was taken as normal ≥40 mg/dl and abnormal< 40 mg/dl

Male participants reported higher levels of education compared to female participants. The majority of participants were married, and the average number of children reported by married women was 5.2 (±2.9). Approximately one-quarter of the currently married females were using some form of contraception, with oral pills being the most commonly reported method (*n* = 343). Approximately 18% (*n* = 542) of women had reached menopause (results not shown).

The average sitting time for a single week day was 360 (±218) minutes (equal to 6.0 h); however, longer durations were reported by the males compared to females (8.0 h vs. 5.0 h, respectively, *p* < 0.01). A weak, but significant negative Pearson’s correlation coefficient value was detected between physical activity and sitting time (r = − 0.16, *p* < 0.001). Bivariate analysis revealed that participants were more likely to have an FRS ≥10 if they were married vs single; if they had an intermediate level of education vs graduate or higher level; or if they were a house wife or worked as a doctor/engineer or were retired vs working as teacher/secretary (Table 3). Family medical history was not shown to be significantly associated with FRS in this study.

**Table 3:** Uni-variable logistic regression analysis between sociodemographic, lifestyle, family history and anthropometric measurements with FRS in Saudi males and females in Riyadh, Saudi Arabia

Variables	Males (*N* = 968)			Females (*N* = 2029)		
	FRS ≥ 10 (*n* = 320)	FRS < 10 (*n* = 648)	Unadjusted odds ratio & 95% CI	FRS ≥ 10 (*n* = 346)	FRS < 10 (*n* = 1683)	Unadjusted odds ratio & 95% CI
*Sociodemographic profile*						
Marital status						
Single	8 (2.5)	94 (14.5)	1.0	4 (1.2)	142 (8.4)	1.0
Married (include widow)^a^	312 (97.5)	554(85.5)	**6.6 (3.1, 13.8)**	342 (98.8)	1541(91.6)	**7.8 (2.8, 21.4)**
Participants Education						
Graduation and above	181 (56.6)	533(82.3)	1.0	39 (11.3)	837 (49.7)	1.0
Intermediate and below	139(43.4)	115(17.7)	**3.5 (2.6, 4.8)**	307(88.7)	846 (50.3)	**7.7 (5.5, 11.0)**
Spouse’s education						
(M = 866,F = 1883)						
Graduation & above	113 (36.2)	361(65.2)	1.0	77 (22.5)	684 (44.4)	1.0
Intermediate and below	199 (63.8)	193(34.8)	**3.2 (2.4, 4.4)**	265(77.5)	857 (55.6)	**2.7 (2.0, 3.6)**
Participants occupation						
Teacher/secretary/ etc.^c^	152(47.5)	471(72.7)	1.0	51(14.7)	717(42.6)	1.0
Military service	32(10.0)	82(12.7)	1.2 (0.7,1.8)	–	–	–
Housewife	–	–	–	266(76.9)	783(46.5)	**4.7 (3.4, 6.5)**
Doctor/Engineer/lawyer^b^	40(12.5)	69(10.6)	**1.8 (1.2,2.7)**	11(3.2)	39(2.3)	**3.9 (1.9, 8.2)**
Unemployed	9 (2.8)	18 (2.8)	1.5 (0.6, 3.5)	5 (1.4)	98 (5.8)	0.7 (0.3, 1.8)
Retired	87 (27.2)	8(1.2)	**33.6 (15.9, 71.3)**	13(3.8)	46(2.7)	**3.9 (2.0, 7.8)**
Spouse’s occupation (M = 866,F = 1883)						
Skilled professions^c^	78 (25.0)	191 (34.5)	1.0	59 (17.3)	559 (36.3)	**1.0**
Military service	–	–		24 (7.0)	250 (16.2)	**4.6 (3.3,6.4)**
Housewife	215 (68.9)	303 (54.7)	**1.7 (1.3, 2.4)**	–	–	**–**
Doctors/Engineer/lawyer^b^	6 (1.9)	27 (4.9)	0.5 (0.2, 1.4)	87 (25.4)	314 (20.4)	**3.3 (1.5, 7.2)**
Unskilled/ unemployed	2 (0.6)	28 (5.1)	**0.1 (0.04, 0.7)**	9 (2.6)	68 (4.4)	0.7 (0.2, 2.0)
Retired	11 (3.5)	5 (0.9)	**5.4 (1.8, 16.0)**	163 (47.7)	350 (22.7)	**4.4 (2.0, 9.2)**
Monthly Income level						
(SAR)^d^						
(M = 943,F = 1718)						
≤10,000	111 (35.9)	209 (33.0)	1.0	159(63.9)	827 (56.3)	1.0
>10,000	198 (64.1)	425 (67.0)	0.9 (0.6, 1.2)	90 (36.1)	642 (43.79)	0.7(0.5, 1.0)
Type of housing^e^						
Arabic style house	16(5.0)	35(5.4)	1.0	32(9.2)	241(14.3)	1.0
Apartment	55(17.2)	286(44.1)	**0.4 (0.2, 0.8)**	37(10.7)	437(26.0)	0.6 (0.4, 1.0)
Villa	249(77.8)	327(50.5)	1.7 (0.9, 3.1)	277(80.1)	1005(59.7)	**2.1 (1.4, 3.1)**
Ownership of house						
Company owned	5 (1.6)	17 (2.6)	1.0	10 (2.9)	73 (4.3)	1.0
Rented	75 (23.6)	273 (42.2)	0.9 (0.3,2.6)	50 (14.5)	449 (26.7)	0.8 (0.4,1.6)
Self-owned	238 (74.8)	357 (55.2)	2.2 (0.8,6.2)	286 (82.7)	1158 (68.9)	**1.8 (0.9,3.5)**
*Anthropometric measurements & Physical activity*						
Body Mass Index (kg/m^2^)^f^						
Normal (< 25)	48 (15.0)	155 (23.9)	1.0	24 (6.9)	266 (15.8)	1.0
Overweight (≥25 to < 30)	120 (37.5)	244 (37.7)	**1.6 (1.1, 2.3)**	81 (23.4)	508 (30.2)	**1.7 (1.1, 2.8)**
Obese (≥30)	152 (47.5)	249 (38.4)	**1.9 (1.3, 2.8)**	241 (69.7)	909 (54.0)	**2.9 (1.8, 4.5)**
Waist Circumference (in cms)^g^						
Normal	114 (35.6)	337 (52.0)	1.0	18 (5.2)	365 (21.7)	1.0
Central obesity	82 (25.6)	149 (23.0)	**1.6 (1.1, 2.3)**	35 (10.1)	359 (21.3)	**1.9 (1.1, 3.5)**
High central obesity	124 (38.8)	162 (25.0)	**2.2 (1.6, 3.1)**	293 (84.7)	959 (57.0)	**6.1 (3.7, 10.1)**
Physical Activity^h^						
High & Moderate	11 (3.4)	77 (11.9)	1.0	127 (36.7)	782 (46.5)	1.0
Low	309 (96.6)	571 (88.1)	**3.7 (1.9, 7.2)**	219 (63.3)	901 (53.5)	**1.5 (1.1, 1.8)**
Sitting time/week day						
(M = 950; F = 2008)						
≤ 6.0 h	83 (26.3)	209 (33.0)	1.0	201 (59.1)	1178 (70.6)	1.0
> 6.0 h	233 (73.7)	425 (67.0)	**1.4 (1.0, 1.9)**	139 (40.9)	490 (29.4)	**1.7 (1.3, 2.1)**
*Family history*						
Family history for Hypertension						
No	194 (60.6)	394 (60.8)	1.0	148 (42.8)	727 (43.2)	1.0
Yes	126 (39.4)	254 (39.2)	1.0 (0.7,1.3)	198 (57.2)	956 (56.8)	1.0 (0.8,1.2)
Family history for Diabetes Mellitus (M = 953,F = 2022)						
No	167 (52.8)	336 (52.7)	1.0	108 (31.3)	634 (37.8)	1.0
Yes	149 (47.2)	301 (47.3)	1.0 (0.8,1.3)	237 (68.7)	1043 (62.2)	**1.3 (1.0, 1.7)**
Family history for CVD						
No	276 (86.3)	555 (85.6)	1.0	279 (80.6)	1382 (82.1)	1.0
Yes	44 (13.8)	93 (14.4)	0.9 (0.6,1.4)	67 (19.4)	301 (17.9)	1.1 (0.8,1.5)
Age of family members with CVD						
No family history	276 (86.3)	555 (85.6)	1.0	279 (80.6)	1382 (82.1)	1.0
Father/ brother age 45 years	20 (6.3)	56 (8.6)	0.7 (0.4,1.2)	29 (8.4)	148 (8.8)	0.9 (0.6,1.5)
Mother/sister age of 55 year	24 (7.5)	37 (5.7)	1.3 (0.7,2.2)	38 (11.0)	153 (9.1)	1.2 (0.8,1.8)

^a^includes currently married, divorced and separated

^b^includes businesspersons, finance manager

^c^Teacher, secretary, health and allied staff, technicians, etc

^e^Arabic type house is a traditional type of house where several families belonging to the same tribe reside

^f^BMI of 18.5–24.9 as normal weight, 25.0–29.9 as overweight and ≥ 30 as obese

^g^High/vigorous physical activity defined as at least 3 days of activity achieving a minimum total physical activity of at least 1500 MET-minutes/week and moderate as “5 or more days of moderate-intensity activity and/or walking of at least 30 min per day” and low activity those not included in high or moderate activity

^h^Waist circumference Normal (Females< 80 cms, Males <90cms; Central obesity (Females > 80 < 88 cms, Males> 90 < 102cms); High central obesity (Females>88cms, Males> 102cms)

^i^sitting time cut-off 6.0 is based on the average sitting time in Saudi population on any weekday

The multivariable logistic regression model, after adjusting for education level and housing type, revealed that the following factors were associated with FRS: *low physical activity* (aOR & 95% CI for males 2.91 (1.45, 5.80) and females 1.38 (1.06, 1.81); *prolonged sitting time* (aOR &95% CI for females 1.58 (1.20, 2.07), and *high central obesity* (aOR& 95% CI for males 2.38 (1.67, 3.41) and females 3.35 (1.92, 5.87). In the males, sitting time > 6 h was marginally associated with FRS (aOR 1.36 (0.98, 1.90). Living in an apartment was protective against a high FRS for males (aOR 0.35, 95%, CI (0.17, 0.71), whereas living in villas, as opposed to Arabic-style housing, was associated with FRS in the females (aOR 2.13 (1.41, 3.24). Participants with a low level of education (intermediate and below) were more likely to have a high FRS (aOR for males 3.49 (2.52, 4.82), females 6.23 (4.31, 8.99), compared to those with a university-level education and higher (Table 4).

**Table 4:** Multivariable logistic regression models** for association of FRS (> 10) with life style factors in Saudi male and females in Riyadh, Saudi Arabia 2014–2015

Variables	Males Odds Ratio (95%CI)	Females Odds Ratio (95%CI)
Physical Activity		
Moderate/high physical activity^a^	1.0	1.0
Low physical activity^b^	2.91 (1.45, 5.80)*	1.38 (1.06, 1.81)*
Sitting time/week day		
Sitting time ≤ 6.0 h	1.0	1.0
Sitting time > 6.0 h	1.36 (0.98, 1.90)	1.58 (1.20, 2.07)*
Waist Circumference^c^		
Normal	1.0	1.0
Central obesity	1.88 (1.29, 2.72)*	1.58 (0.83, 2.99)
High central obesity	2.38 (1.67, 3.41)*	3.35 (1.92, 5.87)*
Type of housing		
Arabic style house^d^	1.0	1.0
Apartment	0.35 (0.17, 0.71)*	0.77 (0.45, 1.29)
Villa	1.46 (0.75, 2.83)	2.13 (1.41, 3.24)*
Participants Education		
Graduation and above	1.0	1.0
Intermediate and below	3.49 (2.52, 4.82)*	6.23 (4.31, 8.99)*

**Models were adjusted for education level and housing type

*values indicate statistically significant with *p* < 0.05

^a^high/vigorous physical activity defined as “at least 3 days of activity achieving a minimum total physical activity of at least 1500 MET-minutes/week” and moderate as “5 or more days of moderate-intensity activity and/or walking of at least 30 min per day” and low activity those not included in high or moderate activity

^b^sitting time cut-off 6.0 was based on the average sitting time in Saudi population on any week day

^c^Waist circumference was defined as Normal (Females< 80 cms, Males <90cms; Central obesity (Females > 80 < 88 cms, Males> 90 < 102cms); High central obesity (Females>88cms, Males> 102cms)

^d^Arabic type house is a traditional type of house where several families belonging to the same tribe reside

## Discussion

The overall ten–year high/intermediate CVD risk (FRS ≥ 10) tended to increase with increasing age in both males and females, but this risk was higher in males compared with females (33.1% vs 17.1%). Previous research determined that men have a greater CVD risk compared with pre-menopausal women, but the risk is similar for post-menopausal women and men [38]. Similar trend was evident in our results, as significant proportion of males had CVD risk in the age category of 30 to 50 years, whereas females were in greater proportion after crossing the age of 50 years. Several explanations related to life, style including physical activity, smoking prevalence, menopause and cholesterol metabolism may help in explaining this variation [39].

The Saudi population has a high prevalence of diabetes mellitus (DM), hypertension (HTN), smoking, hyperlipidaemia, physical inactivity, overweight and obesity [40, 41]. The previous literature is in agreement with our findings, which demonstrated that although the CVD risk tended to be similar in men and women, the individual risk factors differed; for example, smoking is more frequent in men, diabetes mellitus and high-density lipoprotein (HDL) levels are lower in men compared with women, and hypertension is reported to be greater in women aged 65 years or more [42–44].

Apart from age, sex, and family history, most of the above mentioned risk factors are modifiable, and individuals with modifiable risk factors require regular check-ups. It is practical to estimate CVD risk in asymptomatic individuals, using the resulting data to promote knowledge, awareness and as motivation for adopting healthy lifestyles for therapeutic changes [38]. A study from the USA demonstrated a decline in FRS over a period of 15 years among a population undergoing percutaneous intervention and demonstrated a major reduction in the lipid profile and hypertension, whereas risk factors related to body mass index and age increased in the study population [45]. However, even in individuals with moderate or low FRS scores, medical conditions like stroke or acute MI cannot be ruled out, hence it is better to screen them by utilising multiple techniques, in addition to the FRS [45].

European cohort studies showed that FRS underestimated CVD risk in deprived populations and overestimated CVD risk in high risk populations [46, 47]. The NHANES survey data demonstrated a reduction in CVD risk in the US population from 1976 to 1980 to 1988–1994 but less of a reduction from 1988 to 1994 to 1999–2004, particularly in women and middle-aged people [48]. Over the period of thirty years in the USA, changes in risk factors occurred at different rates; and one important contributing factor was an increase in the frequency of obesity over time [48]. Our study revealed that waist circumference, defined as central obesity, was associated with a high FRS in both males and females. The high central obesity in the Saudi population shown in our study implies that this risk is already present in males and females, and any decline in FRS will require tremendous efforts.

Sedentary behaviour and prolonged sitting time are consequences of the social and environmental determinants of urban living; this risk factor awaits interventional investigations to be translated into recommendations [49–51]. A crossover trial demonstrated improved glucose metabolism through brief interruptions of short bouts of light and moderate activity during sitting time [52]. A study from Saudi Arabia conducted in women ≥30 years likewise reported a relationship between increased household activities and strenuous exercise with a reduction in CVD risk [50]. Overall, physical inactivity is high in Saudi Arabia (66.6%), with 87.9% of leisure time spent in physical inactivity [53], especially in elderly, divorced and widowed persons; however, health education has resulted in improvements in lifestyle and physical activity [54]. Our study revealed that a greater percentage of males, compared to females, exhibited a sedentary lifestyle, including low physical activity and prolonged sitting time [8 h vs 5.5 h]. The prospective follow up of > 11 years of a European cohort of men and women revealed changes in the FRS of the participants and demonstrated that those with sedentary lifestyle had the highest risk of CVD [55]. Our findings could potentially be used to devise lifestyle adaptations for improving physical activity, reducing sitting time and addressing adult obesity; such lifestyle changes have been reported to reduce the risk for coronary heart disease (CHD) by 50% [56].

The FRS scores obtained in our study are compatible with local reports of the Saudi population [57] and international populations [58]. The FRS was twice as high for individuals with a first-degree relative who had CVD before the age of 55 in men, and 65 in women [58]. The Saudi study was conducted on 4932 men and women greater than 20 years of age without coronary heart disease, and the results showed that diabetes, dyslipidaemia, and hypertension increased with age [57]. It is evident that the Saudi population has a substantial burden of risk factors, and assessing these risks based upon FRS score in the younger population may not adequately identify individuals who require therapeutic and non-therapeutic interventions [59].

A study from a Scandinavian country reported that apartment living was associated with physical inactivity [60]. However, we obtained contradictory findings wherein residing in an apartment was shown to be protective for males, whereas residing in villas increased the likelihood for females to have a higher FRS. Such diverse findings may be due to operating socio-demographic and environmental factors, which may have undervalued the prevailing risk and require further evaluation. Environmental conditions have been hypothesized to predispose individuals to risky behaviour, and recommendations have been made for testing this hypothesis [48].

Our study has several strengths. First, it was a community based study, conducted in the primary care setting and may accurately represent the Riyadh city population. The FRS has been used in previous studies from Saudi Arabia, and the results are comparable to other standard risk scoring methods [61]. Second, our results provide adequate evidence for the risk factors for CVD and preventive applications to target Saudi males and females for primary and secondary prevention of CVD. Third, our study is one of the few studies that uses sitting time as a predictive factor of FRS in Saudi Arabia, along with physical inactivity. Fourth, a large sample size stratified by age and sex provided comparable estimates that are consistent with other reports on similar topics [42, 48].

However, this study also has certain limitations. This was a cross-sectional study; hence, we could not establish a temporal relationship between the risk factors and CVD risk. We cannot rule out the selection bias that may have occurred for males and females approaching PHCC, as they could be individuals with high FRS scores. Hence our OR estimates could be slightly overestimates; however, there could be other possibility that those with much worse FRS may not have come to PHCC. High accessibility is reported for PHCC for maternal and child health but less accessed for non-communicable diseases [62]. Recall and information biases may have played a role in the reporting of sitting time and physical activity, which may have resulted in misclassification and inaccuracy in these data; however, the use of a physical activity assessment tool has been validated and is reported to underestimate, rather than overestimate, CVD risk [63]. In addition, it is possible that we did not adjust for all potentially confounding variables; thus, residual confounding may have resulted.

Selected cohorts of high-risk populations could be studied using culturally tailored interventions on behavioural and other therapeutic interventions along with other novel markers [64, 65]. We used self-reported physical sitting and activity time, which may be inaccurate; however, it has been validated that self-reported physical activity time attenuates the relationship with FRS scores [63]. PHCCs can serve as multipurpose facility, ranging from screening to bringing changes in life style. A recent report by Ministry of Health refers to use PHCC for promotion of PA in Saudi population [66]. FRS scores obtained in a large population need to be followed by specific algorithms at the population and individual level to adequately address the risks with a two-pronged approach - especially in Saudi populations that exhibit abundant risk factors.

## Conclusion

Females beyond the age of 50 were found to have a higher prevalence for CVD risk and associated factors compared to males of the same age groups. Modifiable risk factors, including prolonged sitting time, increased waist circumference and low physical activity in Saudi men and women from Riyadh have strong implications for primary prevention and management services, which can change the risk profile of the Saudi population. Therefore, culturally appropriate interventions need to be designed and tested.

## Additional file

Additional file 1: Women in Saudi Arabia Health Examination Survey. (DOCX 39 kb)

## Abbreviations

ACE: Africa Middle East cardiovascular epidemiological
BMI: Body mass index
CHD: Coronary heart disease
DM: Diabetes mellitus
FRS: Framingham risk scores
GCC: Gulf countries council
HDL: High density lipoprotein
IPAQ: International physical activity questionnaire
KKUH: King Khalid University hospital
KSA: Kingdom of Saudi Arabia
MET: Metabolic energy expenditure
MI: Myocardial infarction
NHANES: National health and nutrition examination survey
PA: Physical activity
PHCC: Primary health care centres
RACE: Registry for acute coronary events
SD: Standard deviation
SPSS: Statistical package for social sciences
WC: Waist circumference
WISHES: Women in Saudi Arabia health examination survey
