# Supplementary material for: Sedentary lifestyle and Framingham risk scores: a population-based study in Riyadh city, Saudi Arabia
Source: BMC Cardiovasc Disord. 2019 Apr 8;19:88. doi: 10.1186/s12872-019-1048-9 (PMC6454662; doi:10.1186/s12872-019-1048-9)
Supplement: Supplementary file 1 — Women in Saudi Arabia Health Examination Survey. (DOCX 39 kb) [file 12872_2019_1048_MOESM1_ESM.docx]

Women in Saudi Arabia Health Examination Survey

1.1.a. File Number:__________

1.1. b: ID no PHCC Participant number Sex

|  |  |
| --- | --- |

|  |  |  |  |
| --- | --- | --- | --- |

| 2 |
| --- |

| 1.2 | Name: | 1.3 | Date: |
| --- | --- | --- | --- |
| 1.4 | Residence Area :  1. Center of Riyadh  2. North of Riyadh  3. South of Riyadh  4. East of Riyadh  5. West of Riyadh | 1.5 | How you came to know about this research study?   1. Invitation at home 2. Through advertisement at PHCC 3. Through friends/relatives 4. Any other source (specify) |
| 1.6 | Primary Health care center: | 1.7 | Gender 1.Male  2.Female |
| 1.8 | Phone no (mobile): | 1.9 | Phone no (landline in emergency): |
| 1.10. Interviewers name: | | | |

| ***Anthropometric measurements:*** | | | |
| --- | --- | --- | --- |
| 1.11 | Weight(kgs) | 1.12 | Height(cms) |
| 1.13 | Waist circumference(cms) | 1.14 | Hip circumference (cms) |
| 1.15  1.15.a  1.15.b | **Blood pressure(Ist Reading)**  Systolic ______mm Hg  Diastolic________mmHg | 1.16  1.16a  1.16b | **Blood pressure(2nd Reading, after 5 mnts)**  Systolic ______mm Hg  Diastolic________mmHg |

| ***Section: Socio-Demographic data:*** | | | |
| --- | --- | --- | --- |
| ***S.no*** | ***Variable*** | ***Responses*** | ***Explanation for the variable/responses*** |
| 2.1 | **Interview started** | **Exact Time (in Hours: Minutes)** | |
| 2.2 | Age (in years) | Actual age: ___________in years | If not sure about age ask for the Identity card |
| 2.3 | What is your marital status? | 1. Unmarried  2. Married  3. Widow  4. Divorced  5. Separated | 5.Separated means still married but not living with the husband due to marital problems |
| 2.4 | If you are currently married are you | 1. Only wife  2. First wife  3. Second wife  4. Third wife  5. Fourth wife |  |
| 2.5 | What is your Educational level? | 1. Illiterate  2. Primary  3. Intermediate  4. Secondary  5. Diploma  6. University and Postgraduate Studies | 1.Illiterate includes who  -cannot read cannot write  -no formal schooling  5.Diploma means skilled worker with certificate course |
| 2.6 | What is the educational level of your spouse | 1. Illiterate  2. Primary  3. Intermediate  4. Secondary  5. Diploma  6. University and Postgraduate Studies | 1.Illiterate means  -cannot read cannot write  -no formal schooling  5.Diploma means skilled worker with certificate course |
| 2.7 | Participant's occupation | 1. Teacher  2. Secretarial work  3. Business women  4. Sales person  5. Retired  6. Unemployed  7. House wife  8. Other professions \ Specify…… | 3.Business person: owns his/her business  6.Unemployed: eligible and willing to do paid job but has no job  7. House wife: does not do any type of paid job  8.Other Professions: e.g. doctor, lawyer, engineer…etc |
| 2.8 | Spouse's occupation | 1. Teacher  2. Secretarial work  3. Business person  4. Sales person  5. Retired  6. Unemployed  7.Military  8. Other professions \ Specify…… | 3.Business person: owns his/her business  6.Unemployed: eligible and willing to do paid job but has no job  7.Military means person working in army, navy or air force  8.Other Professions: e.g. doctor, lawyer, engineer…etc |
| 2.9 | What type of housing do you live in? | 1. Apartment/flat 2. House (old style house) 3. Villa 4. Palace |  |
| 2.10 | Is your house? | 1. Rented 2. Owned (personally) 3. Company owned |  |
| 2.11 | How much is your total monthly income? | 1. 3000-----5000 SR  2. 5000 ----- 10000 SR  3. 10000---- 20,000 SR  4. More than 20,000  5. Don’t know or don’t want to tell | Monthly income includes income of all members living in the same house |

| ***Section : Medical history*** | | | | | | | | |
| --- | --- | --- | --- | --- | --- | --- | --- | --- |
|  | **Have you been diagnosed by a doctor to have any of the following chronic diseases?** | | | | | |  | |
|  | **Disease** | **(a)**  **Status**  1. Yes 2.No | **(b)**  **Since how long? (years)** | | **(c) Are you currently taking any Medication for this problem?**  1. Yes 2.No 3.dontknow | | **(d) Family History**  1. Yes 2.No  **(Family means immediate family)** | |
| 3.1 | Diabetes mellitus (ie, high blood sugar) |  |  | |  | |  | |
| 3.1.2  a.  b. | If yes, then Type of Diabetes:  Juvenile(childhood diabetes)  Adult onset | | | | | | | |
| 3.1.3  a.  b. | Is it  Insulin dependent  Non-Insulin dependent | | | | | | | |
| 3.2 | Hypertension (ie, high blood pressure) |  | |  | |  | |  |
| 3.3 | Hypothyroidism (ie, low thyroid function) |  | |  | |  | |  |
| 3.4 | Hyperthyroidism (ie, overactive thyroid) |  | |  | |  | |  |
| 3.5 | Osteoporosis (ie, thinning of bones) |  | |  | |  | |  |
| 3.6 | Rheumatoid arthritis  (Identifying symptoms-morning stiffness  -pain in joints  -redness of joints) |  | |  | |  | |  |
| 3.7 | Depression |  | |  | |  | |  |
| 3.8 | Postpartum depression  (depression after delivery upto 1 year) **only ask from Married Females** |  | |  | |  | |  |
| 3.9 | Anxiety |  | |  | |  | |  |
| 3.10 | Celiac disease  (wheat allergy) |  | |  | |  | |  |
| 3.11 | Chronic Diarrhea  (diarrhea lasting for 12 months) |  | |  | |  | |  |
| 3.12 | Stroke |  | |  | |  | |  |
| 3.13 | High Lipids |  | |  | |  | |  |
| 3.14 | Vitamin D deficiency |  | |  | |  | |  |
| 3.15 | Skin diseases |  | |  | |  | |  |
| 3. 16 | Sexually transmitted diseases |  | |  | |  | |  |
| 3.17 | Cardiovascular disease |  | |  | |  | |  |
|  | If yes, for Cardiovascular Disease mention the type | 3.17.1. Heart failure  3.17.2. Myocardial infarction  3.17.3. Angina (recurrent chest pain)  3.17.4. Valvular disease  3.17.5. Transient ischemic attack  3.17.6. Don’t know | | | | | | |
| 3.18 | If Yes for Family History of CVD diseases, specify? | 1. Father or brother before age of 45 years 2. Mother or Sister at age of 55 years or above 3. Both 1 &2 4. I'm not sure 5. Don’t know | | | | | | |
| 3.19 | Have you had any one of the following investigations in the last 2 years? (circle **all** that apply) | 3.19.1.ECG  3.19.2.Echocardiogram  3.19.3.Cardiac catheterization  3.19.4.Stress thallium scan  3.19.5.Exercise tolerance test  3.19.6. None/ I don’t know | | | | | | |
| 3.20 | Are you taking aspirin daily? | 1. Yes  2. No | | | | | | |
| 3.21 | Any other type of chronic disease (liver, kidney, respiratory etc) | 1. Yes 2. No   If yes specify -------------------- | | | | | | |

| ***Section: Smoking related questions*** | | |
| --- | --- | --- |
| 4.1 | Are you currently smoking? | 1.Yes  2.No  ***If No skip to Q 4.6*** |
| 4.2 | Since how long you have been using it? | __________years |
| 4.3 | What do you currently smoke? (Can mark more than one response) | 4.3.1. Sheesha  4.3.2. Tobacco Chewing  4.3.3. Cigarette  4.3.4. Cigar  4.3.5. Others……….. |
| 4.4 | How many cigarettes do you smoke / day? | 1. Less than10 cigarettes  2. 10 to20 cigarettes  3. More than 20 cigarettes |
| 4.5 | If sheesha smoker then how many Hours of a sheesha do you smoke /week? | 1. 5 hours or less  2. 6 – 10 hours  3. 10 – 20 hours  4.More than 20 hours |
| 4.6.a  4.6.b | Have you smoked in the past?  For how many years did you smoke in the past? | 1.Yes  2.No  ***(if No, skip to 4.8)***  1.1-5 years  2. 5-10 years  3.More than 10 years |
| 4.7 | Have you ever smoked =or >100 cigarettes during your life? | 1. Yes  2. No |

| ***Section: Physical Activity – IPAQ (short form)*** | |
| --- | --- |
| READ: I am going to ask you about the time you spent being physically active in the last 7 days. Please answer each question even if you do not consider yourself to be an active person. Think about the activities you do at work, as part of your house and yard work, to get from place to place, and in your spare time for recreation, exercise or sport.    **READ: Now, think about all the *vigorous* activities which take *hard physical effort* that you did in the last 7 days. Vigorous activities make you breathe much harder than normal and may include heavy lifting, digging, aerobics, or fast bicycling. Think only about those physical activities that you did for at least 10 minutes at a time.** | |
| 6.1 | During the **last 7 days**, on how many days did you do **vigorous** physical activities?  1.____ Days per week [VDAY; Range 0-7]  2. Don't Know/Not Sure  3. Refused  [**Interviewer clarification**: Think only about those physical activities that you do for at least 10 minutes at a time.]  [**Interviewer note**: If respondent answers zero, refuses or does not know, skip to Question 6.3] |
| 6.2.a  6.2.b | How much time did you usually spend doing **vigorous** physical activities on one of those days?  1.__ __ Hours per day [VDHRS; Range: 0-16]  2.__ __ __ Minutes per day [VDMIN; Range: 0-960]  3.Don't Know/Not Sure  4.Refused  [**Interviewer clarification**: Think only about those physical activities you do for at least 10 minutes at a time.]  [**Interviewer probe**: An average time for one of the days on which you do vigorous activity is being sought. If the respondent can't answer because the pattern of time spent varies widely from day to day, ask: "How much time in total would you spend **over the last 7 days** doing vigorous physical activities?”  1.__ __ Hours per week [VWHRS; Range: 0-112]  2.___ __ __Minutes per week [VWMIN; Range: 0-6720]  9998. 3. Don't Know/Not Sure  4. Refused |
| 6.3 | **READ: Now think about activities which take *moderate physical effort* that you did in the last 7 days. Moderate physical activities make you breathe somewhat harder than normal and may include carrying light loads, bicycling at a regular pace, or doubles tennis. Do not include walking. Again, think about only those physical activities that you did for at least 10 minutes at a time.**  During the **last 7 days**, on how many days did you do **moderate** physical activities?  1.____ Days per week [MDAY; Range: 0-7]  2. Don't Know/Not Sure  3. Refused    [**Interviewer clarification**: Think only about those physical activities that you do for at least 10 minutes at a time]  [**Interviewer Note**: *If respondent answers zero*, refuses or does not know, skip to Question 5] |
| 6.4.a  6.4.b | How much time did you usually spend doing **moderate** physical activities on one of those days?  1.__ __ Hours per day [MDHRS; Range: 0-16]  2. __ __ __ Minutes per day [MDMIN; Range: 0-960, 998, 999]  3. Don't Know/Not Sure  4.. Refused  [**Interviewer clarification**: Think only about those physical activities that you do for at least 10 minutes at a time.]  [**Interviewer probe**: An average time for one of the days on which you do moderate activity is being sought. If the respondent can't answer because the pattern of time spent varies widely from day to day, or includes time spent in multiple jobs, ask: “What is the total amount of time you spent over the **last 7 days** doing moderate physical activities?”  1.__ __ __ Hours per week [MWHRS; Range: 0-112]  2.__ __ __ __Minutes per week [MWMIN; Range: 0-6720]  3. Don't Know/Not Sure  4.. Refused  READ: Now think about the time you spent walking in the last 7 days. This includes at work and at home, walking to travel from place to place, and any other walking that you might do solely for recreation, sport, exercise, or leisure. |
| 6.5 | During the **last 7 days**, on how many days did you **walk** for at least 10 minutes at a time?  1.____ Days per week [WDAY; Range: 0-7]  2. Don't Know/Not Sure  3. Refused    [**Interviewer clarification**: Think only about the walking that you do for at least 10 minutes at a time.]  [**Interviewer Note**: *If respondent answers zero*, refuses or does not know, skip to Question 7] |
| 6.6.a  6.6.b | How much time did you usually spend **walking** on one of those days?  1.__ __ Hours per day [WDHRS; Range: 0-16]  2. __ __ __ Minutes per day [WDMIN; Range: 0-960]  3. Don't Know/Not Sure  4. Refused  [**Interviewer probe**: An average time for one of the days on which you walk is being sought. If the respondent can't answer because the pattern of time spent varies widely from day to day, ask: “What is the total amount of time you spent walking over **the last 7 days**?”  1.__ __ __ Hours per week [WWHRS; Range: 0-112]  2.__ __ __ __Minutes per week [WWMIN; Range: 0-6720]  3. Don't Know/Not Sure  4. Refused  READ: Now think about the time you spent sitting on week days during the last 7 days. Include time spent at work, at home, while doing course work, and during leisure time. This may include time spent sitting at a desk, visiting friends, reading or sitting or lying down to watch television. |
| 6.7.a  6.7.b | During the last 7 days, how much time did you usually spend ***sitting*** on a **week day**?  1.____ Hours per weekday [SDHRS; 0-16] 2.__ __ __ Minutes per weekday [SDMIN; Range: 0-960]  3. Don't Know/Not Sure  4.. Refused    [**Interviewer clarification**: Include time spent lying down (awake) as well as  sitting]  [**Interviewer probe**: An average time per day spent sitting is being sought. If the respondent can't answer because the pattern of time spent varies widely from day to day, ask: “What is the total amount of time you spent *sitting* last 7 days?”  1.__ __ Hours in one week [SWHRS; Range 0-112]  2.__ __ __ Minutes in one week [SWMIN; Range: 0-6720]  3. Don't Know/Not Sure  4. Refused |

| ***Section: Laboratory investigations*** | | |
| --- | --- | --- |
| **Investigation** | | **Results** |
| 15.6 | Cholestrol (CHOL (mmol/L) |  |
| 15.7 | HDL (mmol/L) |  |
| 15.8 | LDL (mmol/L) |  |
| 15.9 | Triglycerides (TGL(mmol/L) |  |

| **Check list for the Research assistant (please mark “tick √” in front of each completed component)** | |
| --- | --- |
| **Consent** |  |
| **Questionnaire** |  |
| **Anthropometric and Blood pressure measurement** |  |
| **Blood sample** |  |
